# Trauma and suicidality in adolescents with substance use disorders

**DOI:** 10.1186/s13034-026-01165-7

**Published:** 2026-08-30

**Authors:** Mira Vasileva, Sören Kuitunen-Paul, Veit Roessner, Evgenii Shvedovskii, Vera Clemens, Jörg M. Fegert, Yulia Golub

**Affiliations:** 1 Department of Child and Adolescent Psychiatry, Psychosomatic and Psychotherapy, School of Medicine and Health Sciences, Carl von Ossietzky University Oldenburg, Oldenburg, Germany; 2 Clinical Child and Adolescent Psychology and Psychotherapy, Technical University Chemnitz, Chemnitz, Germany; 3 Clinical Psychology and Psychotherapy, Technical University Chemnitz, Chemnitz, Germany; 4 Department of Child and Adolescent Psychiatry, Faculty of Medicine, German Center for Child and Adolescent Health (DZKJ), TU Dresden, Partner Site Leipzig/Dresden, Dresden, Germany; 5 Biological Psychology, Department of Psychology, School of Medicine and Health Sciences, Carl von Ossietzky University of Oldenburg, Oldenburg, Germany; 6 Department of Child and Adolescent Psychiatry/Psychotherapy, University of Ulm, Ulm, Germany

**Keywords:** Trauma, PTSD, Suicidality, Substance use, Adolescents

## Abstract

**Background:**

Substance use and traumatic experiences have been identified as risk factors for suicidal ideation and behavior in community samples. The aim of this study was to explore these associations in a clinical sample of adolescents with substance use disorder (SUD).

**Methods:**

The sample included *N* = 342 adolescents aged 12 to 19 years from a specialized SUD outpatient unit. They reported on their substance use, trauma history, symptoms of posttraumatic stress disorder (PTSD) and depression, and life satisfaction either in a structured clinical interview or standardized questionnaires; they also completed a task assessing inhibitory control. These variables were compared for adolescents with and without suicidal ideation or behavior. Classification and Regression Trees (CART) models were developed to describe subgroups of participants.

**Findings:**

Overall, 34.2% of adolescent SUD outpatients reported suicidal ideation in the last two weeks and 26.0% fulfilled the criteria for suicidal behavior disorder in the last two years according to DSM-5. Female gender (OR = 2.88 and OR = 4.30) and sexual abuse (OR = 4.55 and OR = 5.33) were associated with higher odds of both suicidal ideation and behavior, respectively. Adolescents with suicidal ideation or behavior had significantly more severe SUD-related problems (*d* = -0.46 and *d* = -0.36), symptoms of PTSD (*d* = -1.38 and *d* = -1.04) and of depression (*d*= -1.77 and *d* = − 0.89) and lower life satisfaction (*d* = 0.98 and *d* = 0.81). The CART models identified symptoms of depression and PTSD as the variables that best distinguished participant subgroups.

**Conclusions:**

These results indicate high rates of suicidal ideation and behavior in adolescents with SUD and underline the importance of assessing trauma when identifying and preventing suicidality in this population.

**Supplementary Information:**

The online version contains supplementary material available at https://doi.org/10.1186/s13034-026-01165-7.

Suicidal ideation and suicidal behavior as well as substance use all peak in adolescence [1, 2]. Furthermore, these mental health problems co-occur, often in the context of trauma, and are associated with poorer outcomes, including repeated suicide attempts, chronicity, school dropout, or interpersonal problems [3, 4]. This represents a serious public health challenge and emphasizes the need to extend our knowledge about the prevalence and mechanisms behind this co-occurrence in order to better detect and prevent both, suicidality and substance use, in adolescents.

For this study, we adopted selected aspects of the nomenclature for suicidality proposed by Silverman and colleagues [5]. Specifically, we incorporated the criterion of intention to die while excluding lethality, because participants retrospectively reported on suicidality. Suicidality was classified as (1) suicidal ideation (thoughts such as “I’m thinking about killing myself”), ranging from passive wishes for death to active consideration or planning of a suicide attempt, and (2) self-intended suicidal behavior referring to suicide attempts. To use a clinically relevant definition, we specified suicide attempts as “a self-initiated sequence of behaviors by an individual who, at the time of initiation, expected that the set of actions would lead to his or her own death” as defined within the criteria for suicidal behavior disorder in the Diagnostic Classification of Mental Disorders (DSM-5) [6].

The global prevalence of suicidal ideation in 13- to 15-year-olds has been estimated at 15% to17% and for suicidal behavior at 17% [7, 8]. Results from community samples, both in adults and adolescents, identified substance use as an important risk factor for suicidality [9–11]. For instance, a recent meta-analysis including 47 studies indicated that harmful substance use was associated with fivefold higher risk for suicidal behavior [11]. Furthermore, substance use (especially in females) was associated with a higher risk for multiple suicide attempts [9]. In adolescent community samples, female adolescents with a history of suicidal behavior showed sixfold higher risks for substance use disorder (SUD) [3].

Substance use and its clinically relevant patterns such as chronic or polysubstance use have been operationalized differently in community samples leading to different effect sizes of the associations with suicidality [11]. Adolescents who seek substance-use-related treatment usually fulfill the criteria for a SUD including acute intoxication, harmful use, and dependence syndrome according to ICD-10 [12]. Lifetime prevalence of SUD in adolescents was estimated at 11.4% and 12-month prevalence at 4.5% to 8.3% [4].

A common risk factor for suicidality as well as SUD is trauma history in the presence or absence of post-traumatic stress disorder (PTSD). Previous studies have highlighted that traumatic experiences, especially interpersonal trauma, and symptoms of PTSD were respectively three and five times more common in adolescents with SUD, with trauma often occurring before the first use as a coping attempt [13, 14].

From a theoretical perspective, the Interpersonal Theory of Suicide [15] provides a useful framework for understanding the interaction between suicidality, substance use, and trauma. According to this model, suicidal desire arises from thwarted belongingness and increased perception of being a burden. Such thwarted belongingness may be intensified by trauma exposure and co-occurring psychiatric symptoms frequently observed in adolescents with SUD. In addition, the acquired capability for suicide may increase the likelihood that suicidal ideation progresses to suicidal behavior and in this way act as a moderator. This acquired capability can develop through repeated exposure to painful and fear-inducing experiences such as sexual or physical abuse or high-risk behaviors.

The theory also proposes that genetic and temperamental predispositions to relevant factors such as impulsivity defined as the predisposition to immediately act to internal or external stimuli can make individuals more susceptible to acquiring the capability for suicide [15]. Recent research has also highlighted the importance of temperament, affect regulation, and neurocognitive mechanisms in shaping suicidal behavior [16]. On the other hand, self-control including inhibitory control – the ability to suppress impulsive or maladaptive responses – has been seen as the neurocognitive counterpart to impulsivity. Poor self-control was found to be linked to a higher likelihood of transitioning from suicidal ideation to suicide attempts even when controlled for impulsivity [17].

Although both SUD and trauma were found related to suicidality in community samples, little is known about suicidal ideation and behavior specifically in adolescents with SUD. There is only one study to our knowledge with a clinical sample of adolescents with SUD investigating the rates of and factors associated with suicidality: A study of 503 adolescents with SUD found 17% reporting lifetime suicidal behavior with co-occurring psychiatric disorders, particularly mood disorders, showing the highest associations of the factors investigated within this study [18].

In light of the scarce research of adolescents with SUD and co-occurring suicidal ideation and behavior, this study aimed to exploring factors related to suicidal ideation and behavior in adolescents with SUD (i.e., gender identity, substance use, trauma history, co-occurring psychiatric disorders, inhibitory control). Furthermore, adolescents with and without suicidal ideation and behavior were compared in terms of their substance use history and problems, symptoms of PTSD, symptoms of depression, life satisfaction and inhibitory control. In line with the exploratory design of the study, the analyses are intended to identify potential associations and inform future confirmatory research.

## Methods

### Participants and procedure

Participants (*N* = 390) were recruited from 2017 to 2024 in a specialized SUD outpatient unit in Dresden, Germany. All adolescents presenting to the clinic, were informed about the study by their therapists and asked to participate. Written consent was provided by each participant and their legal guardian. Study design and procedures were approved by the ethics commission of the University Hospital C. G. Carus Dresden (EK 66022018). The study was designed to evaluate a SUD treatment program (DELTA [19]). The assessment took place at approximately four appointments. Adolescents were included if they were between 12 and 18 years of age and fulfilled the criteria for SUD according to ICD-10. Exclusion criteria included pre-existing neurological disorders, somatic diseases involving the central nervous system (CNS), an IQ below 70, disorders of the adrenal glands, pituitary gland, or hypothalamus, acute viral or bacterial illness, and refusal of study participation by either parents or adolescents. Current substance use was not an exclusion criterion. The present manuscript reports a secondary analysis of the baseline data. From the original sample of *N* = 615 adolescents, we selected those with SUD and available data on suicidal ideation and behavior (*N* = 390). Demographic characteristics of the participants are summarized in Table 1. The sample included 42% female participants. Most adolescents reported German nationality and were living with their biological parents. Half of the adolescents, who underwent a medical examination (*n* = 83), reported non-suicidal self-injurious behavior (NSSI). Rates of SUD are displayed in Supplementary Table 1. Due to item-level missing data, two separate analytic samples were formed depending on the outcome variable. For suicidal ideation, complete data were available for *n* = 342 participants, whereas for suicidal behavior complete data were available for *n* = 196 participants. Thus, analyses for suicidal ideation and suicidal behavior were conducted in partially overlapping but not identical subsamples.

**Table 1:** Sample demographic characteristics (*N* = 390)

Variable		*n* (%)
Age (*M* (*SD*))		16.03 (1.3)
Gender		
Male		224 (57.7%)
Female		164 (42.3%)
Nationality (*n* = 277)		
German		272 (98.2%)
Other		5 (1.8%)
Living with… (*n* = 273)		
Mother		114 (41.8%)
Mother and father		48 (17.6%)
Stationary facility		38 (13.9%)
Father		12 (4.4%)
Therapy Facility		8 (2.9%)
Other		53 (19.4%)
Non-Suicidal Self-injurious Behavior (medical examination), *n* = 83		43 (52%)

### Measures

#### Suicidal ideation and depression

Symptoms of depression and suicidal ideation in the past two weeks were assessed using the Beck Depression Inventory II (BDI-II) [20], a 21-item self-report measure. Each item refers to the last two weeks, is rated on a Likert scale from 0 to 3, with higher total scores indicating greater severity of symptoms of depression. Suicidal ideation was classified as present when a score of ≥ 1 was recorded on item 9 (0 = “I don’t have any thoughts of killing myself”; 1 = “I have thoughts of killing myself, but I would not carry them out”; 2 = “I would like to kill myself”; and 3 = “I would kill myself if I had the chance.“). For the purpose of this analysis, we used the total score without suicidal ideation in order to avoid overlapping variables. In the current sample, internal consistency was high (α = .94).

#### Suicidal behavior

The Mini-International Neuropsychiatric Interview for Children and Adolescents (MINI-KID [21]) was utilized to evaluate psychiatric diagnoses in adolescent patients. A trained clinical psychologist administered the interview. For this study, we used the categorization of suicidal behavior disorder in the last 24 months according to the DSM-5 criteria.

#### Child and adolescent psychiatric diagnosis

The diagnoses of current mental disorders were made according to the ICD-10 criteria based on routine clinical assessment by staff members of the specialized outpatient clinic for SUD, including physicians in training, psychotherapists, and psychologists. This assessment included determining whether participants met the ICD-10 criteria for SUD. The diagnosed SUDs included mental and behavioral disorders due to alcohol (F10.X), opioids (F11.X), cannabinoids (F12.X), sedatives or hypnotics (F13.X), cocaine (F14.X), other stimulants, including caffeine (F15.X), hallucinogens (F16.X), volatile solvents (F18.X), and multiple substance use as well as use of other psychoactive substances (F19.X). For the present analysis, only diagnoses F1X.1 harmful use and F1X.2 dependence were considered. We did not include tobacco for the purpose of this study. Using a structured interview guide, all six SUD symptoms were assessed, and diagnoses of harmful use (fewer than three symptoms) and dependence (three or more symptoms) were assigned. For this analysis we also created a variable indicating the presence or absence of co-occurring psychiatric disorders besides SUD (if the criteria for at least one further disorder in ICD-10 were fulfilled).

#### Non-suicidal self-injurious behavior

To better describe the sample, we reported NSSI; however, this was not included as an outcome variable, as the present study focused on suicidal behavior [5]. As part of the physical examination, data on NSSI were obtained through a combination of patient non-standardised self-report and clinical observation. Participants were asked whether they had ever engaged in self-injurious behaviors without suicidal intention (e.g., cutting, burning, or hitting objects), corresponding to a lifetime assessment. To distinguish NSSI from accidental injuries, only behaviors explicitly described as intentional self-harm were coded as NSSI. Cases without visible physical findings (e.g., no scars or marks) were still classified as NSSI if self-reported. In addition, visible physical consequences such as scars were documented during the clinical examination at the discretion of the examining physician.

#### Substance use history

A structured questionnaire [19] developed for the purposes of this study assessed the frequency and the average amount consumed per day for the following substances over the past month and the past year: tobacco, alcohol, cannabis, methylenedioxymethamphetamine (MDMA), amphetamines, methamphetamines, cocaine, hallucinogens, ketamine, opiates, and other substances. The variables included age at first use, trial use (frequency in number of consumption days), amount per day, number of consumption days per month, average daily amount, route of administration (oral, nasal, rectal, intravenous), and subjective effects. It was developed based on clinical expertise and the specific needs of the specialised outpatient clinic and was administered by the clinician.

#### Severity of substance use

The Drug Use Disorders Identification Test (DUDIT [22]), in its German adaptation provided by the EMCDDA, is a self-administered questionnaire consisting of 11 items designed to detect the severity of substance use (e.g., “How often over the past year have you taken drugs and then neglected to do something you should have done?”). The scoring system is divided into two parts: items 1 through 9 use a five-point Likert scale, whereas items 10 and 11 are rated on a three-point scale with possible scores of 0, 2, and 4, respectively. The total DUDIT score is obtained by summing all item responses, with the highest possible score of 44. Previous studies have shown good psychometric properties of the DUDIT for the use with adolescents [23]. Cronbach’s Alpha in the current sample was α = 0.87.

#### Trauma history and symptoms of PTSD

The University of California at Los Angeles Post Traumatic Stress Disorder Reaction Index for DSM-IV (UCLA PTSD [24, 25]) is a self-report tool for screening for potentially traumatic exposure and symptoms of PTSD. The instrument includes a trauma history section where individuals identify the traumatic events that have impacted them. For the purposes of this study, we analysed interpersonal traumatic events (physical abuse, family violence, community violence, sexual abuse, physical neglect). The questionnaire also assesses the frequency of PTSD symptoms according to DSM-IV experienced in the past month (e.g., “I have upsetting thoughts or images about what happened that pop into my head.”), rated on a scale from 0 (none of the time) to 4 (most of the time). A total PTSD symptom score was used in this study to indicate severity of symptoms of PTSD. The internal consistency in the current sample was α = 0.93.

#### Life satisfaction

Adolescents rated their global life satisfaction on the Satisfaction with Life Scale (SWLS), German version [26]. This is a 5-item questionnaire rated on a 7-point Likert scale with higher scores (max. 35) indicating higher life satisfaction (e.g., “In most ways, my life is close to my ideal.”). Cronbach’s Alpha in the current sample was α = 0.85.

#### Inhibitory control

The Test of Attentional Performance (TAP [27]) was employed to evaluate cognitive functions associated with attention and response inhibition. Specifically, the “Go/NoGo” task was applied to measure inhibitory control. In the TAP’s “Go/NoGo” task, participants were presented with a target stimulus (Go trial) that required a prompt response and a similar but non-target stimulus (NoGo trial) that called for response suppression, thereby providing a measure of inhibitory control. To aid interpretation, an inverse efficiency score (IES) was calculated, with higher IES values indicating poorer inhibitory control (IES = mean reaction time of correct responses / proportion of correct responses to trials).

### Statistical analysis

Analyses were conducted in IBM SPSS 30 and R (version 4.5.2). Missing data were imputed on scale level using the expectation-maximization algorithm for participants with at least 50% available data on a given scale. This decision was made in order not to include completely imputed data that might not have clinical relevance. Little’s test indicated data missing completely at random for the scale SLWS (*p* > .05) but not for BDI, UCLA, and DUDIT (*p* < .05). Since no patterns of missingness could be observed when more precisely examining the data, we assumed the data to be missing at random (MAR). For most participants (92.1%-98.3% depending on the scale of interest) at most one item was imputed. No data were imputed for suicidal ideation and behavior. First, we reported proportions for suicidal ideation and behavior. Next, adolescents were categorized into groups: with/without suicidal ideation based on the BDI-II item about suicidal ideation; and with/without suicidal behavior based on the MINI-KID results. Chi-square tests and odds ratios were used to examine the associations of the group membership with gender identity, type of SUD, traumatic experiences, and psychiatric disorders. Furthermore, the two sets of groups were compared regarding current age, age at first use, symptom severity of depression as well as PTSD, life satisfaction, and inhibitory control. Interactions with gender and each independent variable were explored in logistic regression models separately for suicidal ideation and suicidal behavior. In each model, gender and either type of SUD, traumatic experiences, or psychiatric disorders as well as the interaction term between gender and the variable of interest were simultaneously entered as independent variables.

Finally, we employed Classification and Regression Trees (CART) using the rpart package in R to model the dichotomous outcomes – suicidal ideation and behavior. This method allows for a non-parametric, recursive partitioning approach that identifies subgroups of participants based on associated variables and their interactions, producing an interpretable decision tree. The algorithm recursively splits the data into homogeneous subsets by selecting associated variables and split points that maximized reduction in impurity, measured via the Gini index for classification. As the primary aim of the CART analyses was exploratory and descriptive, the resulting trees were intended to identify potentially relevant subgroup structures and interactions rather than to establish predictive models for clinical application. Model complexity was controlled through the complexity parameter, which was optimized via 10-fold cross-validation to prevent overfitting. The final tree structure was visualized using the rpart.plot package. Terminal node sample sizes are displayed in the figures to facilitate interpretation. Each node represents a subgroup of participants defined by the decision rules of the CART model. Values within nodes indicate the class probabilities for the outcome categories (e.g., no suicidal ideation vs. suicidal ideation). Terminal nodes (end nodes) represent the final subgroups identified by the model, for which no further splitting was performed. Percentages shown only in terminal nodes indicate the proportion of the total sample classified into each final subgroup.

## Results

In the current sample, *n* = 117 (34.2%) adolescents reported suicidal ideation in the last two weeks. A total of *n* = 51 (26.0%) fulfilled the criteria for suicidal behavior disorder in the last two years.

**Fig. 1:**
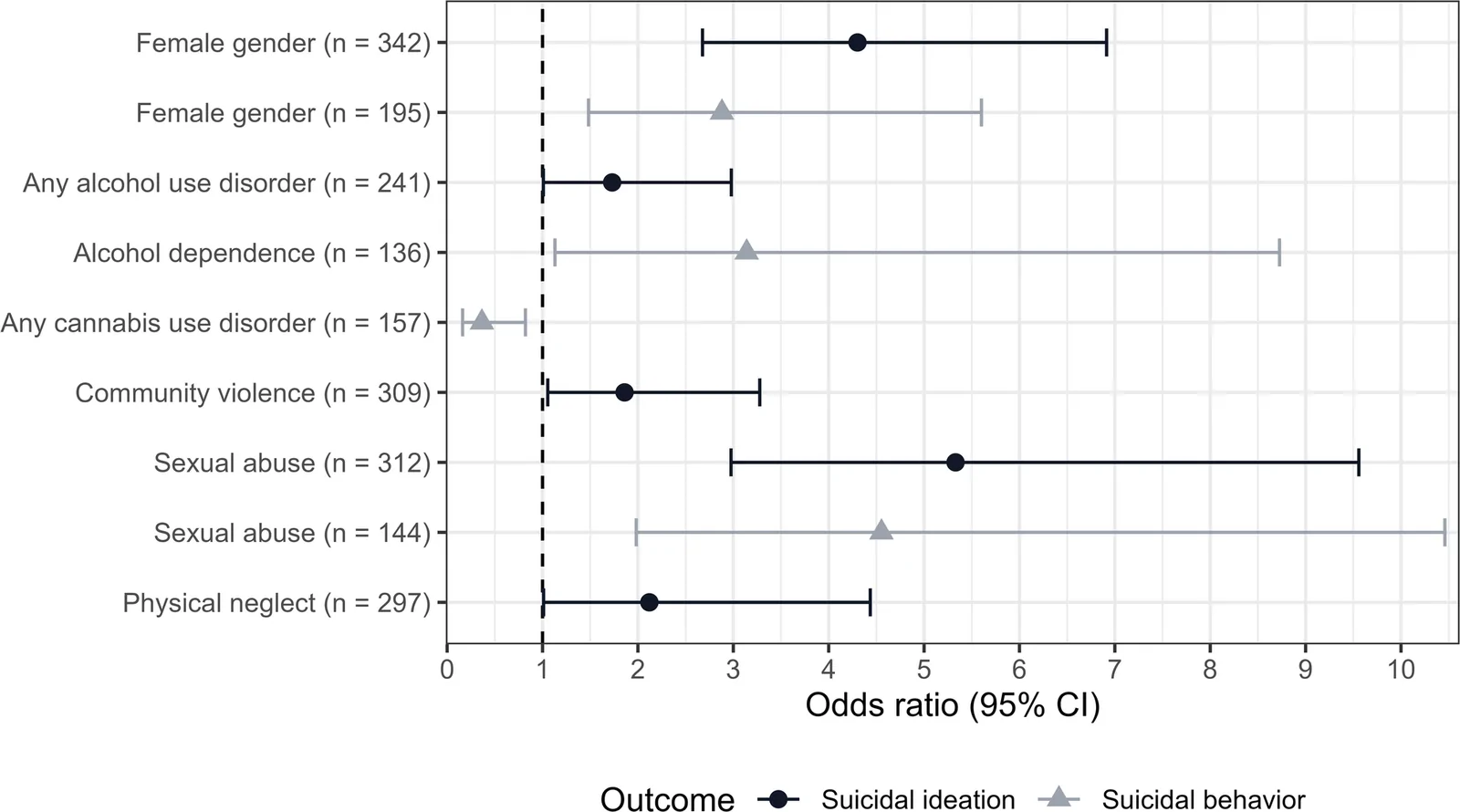
Odd ratios and 95% confidence intervals for variables significantly associated with suicidal ideation and behavior in the dichotomous analysis. Sample sizes differ due to data availability

Variables significantly associated with suicidal ideation or behavior are shown in Fig. 1 (see Supplementary Table 2 for a detailed overview). Female participants had significantly higher rates of suicidal ideation or behavior (χ^2^ = 38.40, *p* < .001, OR = 4.30, 95% CI [2.68–6.91], *n* = 342; χ^2^ = 10.11, *p* < .001, OR = 2.88, 95% CI [1.48–6.61], *n* = 195). Any alcohol SUD as well as alcohol dependence were variables significantly associated with suicidal ideation (χ^2^ = 3.99, *p* < .046, OR = 1.73, 95% CI [1.01–2.97], *n* = 241) and suicidal behavior (χ^2^ = 5.16, *p* = .023, OR = 3.14, 95% CI [1.13–8.73], *n* = 136), respectively. Any cannabis SUD was significantly associated with lower odds of suicidal behavior (χ^2^ = 6.20, *p* = .013, OR = 0.36, 95% CI [0.16–0.82], *n* = 157).

There were some patterns of interpersonal trauma exposure that differed for suicidal ideation and behavior: Adolescents who reported community violence (χ^2^ = 4.67, *p* = .031, OR = 1.86, 95% CI [1.06–3.28], *n* = 309) and physical neglect (χ^2^ = 4.09, *p* = .043, OR = 2.12, 95% CI [2.12–4.44], *n* = 297) were more likely to have reported suicidal ideation. Adolescents who reported sexual abuse were more likely to have reported both suicidal ideation (χ^2^ = 35.25, *p* < .001, OR = 5.33, 95% CI [2.98–9.56], *n* = 312) and suicidal behavior (χ^2^ = 13.91, *p* < .001, OR = 4.55, 95% CI [1.92–10.46], *n* = 144). There were no significant associations regarding stimulant-related SUD, any co-occurring psychiatric disorders besides SUD, and family violence (all *p* > .05).

Furthermore, adolescents with suicidal ideation or behavior showed significantly lower life satisfaction and significantly higher SUD-related problems, symptoms of depression and PTSD (Table 2). Regarding the occurrence of index trauma, onset of PTSD or inhibitory control, there were no significant differences regarding current age, age at first use, age at trauma or PTSD onset (all *p* > .05).

**Table 2:** Mean differences of age, life satisfaction, and PTSD as well as depression symptoms in adolescents with and without suicidal ideation or behavior

Variables (*n*_1_; *n*_2_)^a^		Suicidal ideation					Suicidal behavior				
		M (SD)		t (df)	*p*	Cohen’s d	M (SD)		t (df)	*p*	Cohen’s d
		No	Yes				No	Yes			
Current age (*n*_1_ = 342, *n*_2_ = 196)		16.09 (1.27)	16.05 (1.19)	0.57 (340)	0.570	-	15.94 (1.21)	15.95 (1.26)	-0.69 (194)	0.945	-
Age at first use^b^ (*n*_1_ = 271, *n*_2_ = 157)		14.09 (1.20)	13.89 (1.14)	1.31 (269)	0.096	-	14.07 (1.33)	13.77 (1.25)	1.32 (155)	0.187	-
Age index trauma (*n*_1_ = 112, *n*_2_ = 55)		14.29 (11.67)	11.98 (4.11)	1.30 (110)	0.098	-	14.64 (11.02)	12.94 (3.57)	0.60 (53)	0.549	-
Age beginning of PTSD (*n*_1_ = 100, *n*_2_ = 52)		11.93 (3.73)	11.93 (3.65)	-0.07 (85)	0.473	-	11.92 (4.07)	12.13(3.76)	− 0.18 (50)	0.861	-
Severity substance use (*n*_1_ = 326, *n*_2_ = 151)		15.67 (19.29)	20.49 (11.06)	-3.90 (324)	< 0.001	-0.46	15.51 (9.61)	19.09 (11.14)	-1.89(149)	0.030	-0.36
Life satisfaction (*n*_1_ = 322, *n*_2_ = 153)		22.08 (6.49)	15.73 (6.56)	8.29 (320)	< 0.001	0.98	21.42 (6.91)	15.87 (6.63)	4.29(151)	< 0.001	0.81
Symptoms of PTSD (*n*_1_ = 215, *n*_2_ = 112)		22.09 (14.11)	43.06 (16.76)	-9.87 (213)	< 0.001	-1.38	21.34 (14.28)	38.46 (16.65)	-5.23(110)	< 0.001	-1.04
Depression^c^ (*n*_1_ = 344, *n*_2_ = 155)		10.78 (8.23)	28.28 (12.44)	13.73 (170)	< 0.001	-1.77	14.28 (11.13)	25.64 (14.11)	-4.73 (153)	< 0.001	-0.89
Inhibitory control (*n*_1_ = 123, *n*_2_ = 84)		500 (134)	502 (99)	-0.08 (121)	0.938	-	495 (82)	492 (112)	0.15 (82)	0.881	-

^a^The *n*_*1*_ for the analyses of suicidal ideation and *n*^*2*^ for the analyses of suicidal behavior for the comparisons may differ due to data availability

^b^For any substances except tobacco

^c^Excluding the item 9 about suicidal ideation

Significant interactions were found between gender and any cannabis SUD and cannabis dependence on suicidal ideation (*B* = 1.54, *SD* = 0.68; *p* = .025, *n* = 240) and behavior (*B* = 1.89, *SD* = 0.92; *p* = .041, *n* = 136), respectively (Supplementary Table 3). For both significant interactions, the proportion of suicidal ideation or behavior was higher for females with cannabis-related SUD compared to males with cannabis-related SUD (Supplementary Fig. 1). No further interactions between gender and the other examined variables in this study were significant (*p* < .05).

**Fig. 2:**
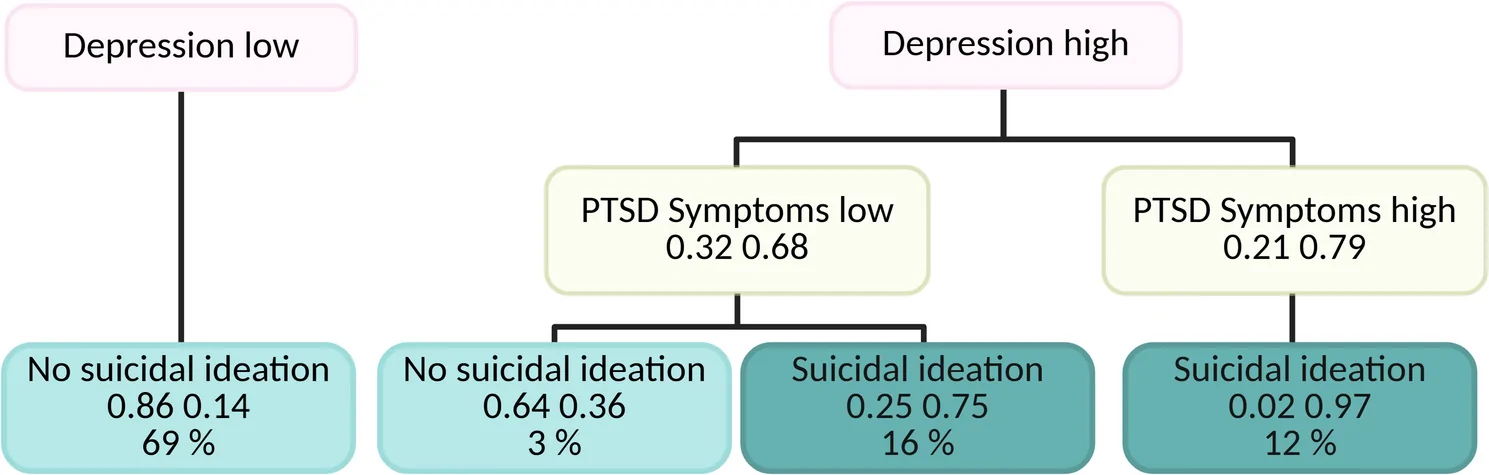
CART model for suicidal ideation (Splitting points identified by the model: BDI = 23; UCLA = 25); Numbers inside nodes show predicted class probabilities. Terminal nodes represent final subgroups. Percentages indicate the proportion of the total sample in each terminal node

**Fig. 3:**
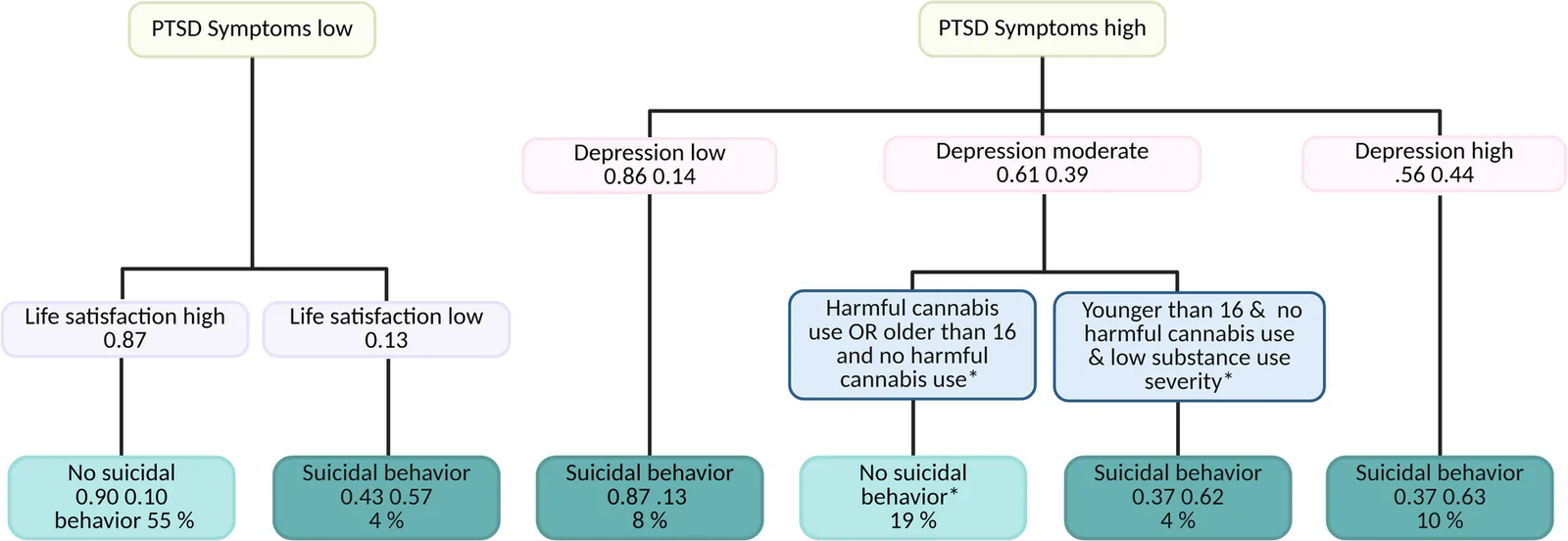
CART model for suicidal behavior (Splitting points identified by the model: UCLA = 30; SWLS = 14; BDI = 13734; DUDIT = 2; Numbers inside nodes show predicted class probabilities; Terminal nodes represent final subgroups. Percentages indicate the proportion of the total sample in each terminal node; *Subgroups are descriptively combined to facilitate interpretation

The CART model identified more severe symptoms of depression and of PTSD as the most important factors for suicidal ideation (Fig. 2). For suicidal behavior, subgroups were best categorized by symptoms of PTSD, depression, and life satisfaction (Fig. 3).

## Discussion

The aim of this study was to provide deeper insight into suicidal ideation and behavior among adolescents with SUD, a population that has been understudied to date. A particular strength of the study is the substantial proportion of female patients (42.3%) with SUD in our sample. Adolescents with SUD descriptively showed higher rates of suicidal ideation and behavior compared to the general population [28]. Along with known associated factors (i.e. history of sexual abuse, more severe symptoms of depression and PTSD, low life satisfaction), we found few substance-specific factors related to suicidal ideation and behavior – such as more severe substance use, higher rates of alcohol-related SUD, and lower rates of cannabis use disorder.

Our results highlight that adolescents with SUD and suicidal ideation or behavior show high mental health burden characterized by more severe mental health symptoms and lower life satisfaction. These results are in line with previous studies both with a clinical sample of adolescents with SUD [18] and samples from the general population [9] showing that depression and trauma are associated with suicidal ideation and suicidal behavior. Although we examined the association between suicidal ideation and depressive symptoms as measured by the BDI excluding the item on suicidal ideation, the conceptual overlap could not be fully eliminated because suicidal ideation and suicidal behavior are considered symptoms of depression in current diagnostic classifications [6, 12]. However, it should also be acknowledged that suicidal ideation and behavior are not specific to depression and may occur in the presence of other psychiatric disorders or even in their absence [7].

The results from the CART model, identifying variabes that best describe subsamples of adolescents with SUD characterized by suicidal ideation or behavior, can be interpreted in the context of theoretical frameworks existing for more general populations. In our CART model but also reflected in the dichotomous analysis, we found that depression was particularly important for suicidal ideation while symptoms of PTSD and low life satisfaction played a more prominent role for suicidal behavior. Similar to the factors we identified in adolescents with SUD, theoretical models such as the Interpersonal Theory of Suicide [15, 29] also explain suicidal behavior through the interplay between symptoms of depression and low life satisfaction that might be related to thwarted belongingness, and trauma history, which might increase the capability for suicide. However, we did not find an association between low inhibitory control and suicidal behavior as expected based on previous findings [17]1. It should be noted that theoretical considerations propose that not only self-control but rather high impulsivity leads to actual suicide attempts [15, 29]. We were not able to control for impulsivity in the current study. Hence, future studies need to further investigate the role of impulsivity as well as broader or other operationalization of self-control.

One of the strongest associations in our study was found for history of sexual abuse and both suicidal ideation and behavior. In the current sample, there was a very high rate of sexual abuse, especially for females (20% in the whole sample, 44% in female participants). Existing literature has repeatedly demonstrated the link between sexual abuse and substance use in females [30]. It is important to consider that females may face additional (re-)traumatization within drug-using environments or even within addiction treatment settings, which are often male-dominated [31, 32]. These settings may not adequately address the specific needs of female adolescents [33]. Therefore, the implementation of gender-specific or gender-sensitive interventions is essential, as the underlying mechanisms such as trauma exposure might differ between male and female adolescents.

There were few associations between specific SUD and suicidal ideation and behavior which might be explained with the fact that most adolescents in the sample reported polysubstance use. These associations should be interpreted cautiously given the small effect sizes, limited sample sizes within subgroups, and the heterogeneity of the comparison groups. We found that alcohol dependence compared to other SUD was more often related to suicidal behavior. However, this finding should be interpreted with caution given the very small number of adolescents fulfilling criteria for alcohol dependence in the present sample (*n* = 8). A previous study found that shared genetic liability for alcohol consumption, alcohol problems, and suicidal behavior was related to higher impulsivity in adults, which in turn increases the risk for suicidal behavior [34]. Due to the small number of adolescents with alcohol dependence, it is necessary to replicate the findings in a larger sample.

We found significantly lower odds for suicidal behavior in adolescents with cannabis use disorder compared to adolescents with other SUDs. This finding should not be interpreted as indicating a protective effect of cannabis use. In contrast, in the general population, cannabis use (vs. no history of cannabis use) has been found to increase the risk for suicidal behavior [35]. However, here we had as a comparison group adolescents with other SUD instead of non-users, so the observed association reflects differences within a clinically affected population and does not suggest lower suicidality risk relative to adolescents without substance use problems. A conceivable hypothesis is that adolescents with cannabis use disorder might constitute a subgroup within clinical populations who are less psychiatrically burdened than peers with other or multiple SUD. For females, this association was weaker. The interaction between gender and cannabis use suggests that targeted interventions for females with cannabis use disorders could be crucial in reducing suicidality risk. However, here the effect was also small and the comparison groups heterogeneous, which limits the generalizability of this finding.

There are several limitations of the current study. We analyzed cross-sectional data which precludes conclusions regarding temporal relationships or causality. The sample sizes for suicidal ideation and suicidal behavior as well as the assessment methods and time frames (two weeks versus two years) were different which hampers comparability. The bivariate analyses were not corrected for multiple testing which inflates the Type I error. Although we emphasized on describing subgroups of adolescents with suicidal ideation or behavior rather than concluding prediction, some of the statistical models (regression, CART) were developed for prediction modelling and have to be interpreted with caution. The MINI- KID interview does not ask the age of the first suicide attempt which hampered studying the temporal relationship between the onset of suicidal behavior and substance use. The assessment of suicidal behavior using one BDI item did not allow a comprehensive and differentiated evaluation of this construct. By excluding the item about suicidal ideation from BDI, our results (e.g., mean and standard deviations) cannot be directly compared with other studies using the full BDI score. Furthermore, we did not analyse further related outcomes such as non-suicidal self-injury. Dichotomizing psychiatric comorbidity may mask important differences related to the type of co-occurring disorders as well as the number of comorbidities.

Despite these limitations, there are some important implications of our findings for the clinical practice and future research. Our results underscore the elevated risk of suicidal ideation and behavior in adolescents with SUD, particularly emphasizing the high mental health burden. These findings may support the value of routine screening for suicidal ideation and behavior in adolescents with SUD, with special attention to trauma history, particularly sexual abuse, and co-occurring psychiatric symptoms. The observed gender-specific associations suggest that gender-sensitive prevention and intervention strategies may be beneficial and deserve further investigation. Furthermore, longitudinal studies are needed to elucidate the temporal relationships and causal pathways linking trauma, mental health symptoms, substance use patterns, and suicidal ideation and behavior in adolescents with SUD.

## Electronic Supplementary Material

Below is the link to the electronic supplementary material.

Supplementary Material 1.

## Data Availability

The data that support the findings of this study are not publicly available due to ethical restrictions to protect participant privacy. he R code used for the analyses is available from the corresponding author upon reasonable request.
