# Supplementary material for: Trauma and suicidality in adolescents with substance use disorders
Source: Child Adolesc Psychiatry Ment Health. 2026 Aug 30;20:121. doi: 10.1186/s13034-026-01165-7 (PMC13548469; doi:10.1186/s13034-026-01165-7)
Supplement: Supplementary file 1 [file 13034_2026_1165_MOESM1_ESM.docx]

**Supplementary Table 1**

Substance use disorders according to ICD-10 (*N* = 390)

| **Variable** | | ***n* (%)** |
| --- | --- | --- |
| ICD-10 SUD Alcohol | |  |
|  | Harmful use | 94 (24.1%) |
|  | Dependence | 34 (8.7%) |
|  | Any | 139 (35.6%) |
| ICD-10 SUD Cannabinoids | |  |
|  | Harmful use | 100 (25.6%) |
|  | Dependence | 95 (24.4%) |
|  | Any | 213 (54.6%) |
| ICD-10 SUD Stimulants | |  |
|  | Harmful use | 62 (15.9%) |
|  | Dependence | 38 (9.7%) |
|  | Any | 109 (27.9%) |
| Polysubstance use (SUD incl. more than one substance, excluding tobacco) | | 171 (43.8) |

*Note.* SUD are displayed if >10% in the total sample

**Supplemetary Table 2**

Group comparison between adolescents with and without suicidal ideation or behavior

| **Variables** | | **Suicidal ideation** | | | | | **Suicidal behavior** | | | | |
| --- | --- | --- | --- | --- | --- | --- | --- | --- | --- | --- | --- |
|  |  | ***n* (%)** | | **χ^2^** | ***p*** | **OR** | ***n* (%)** | | **χ^2^** | ***p*** | **OR** |
|  |  | **No** | **Yes** |  |  |  | **No** | **Yes** |  |  |  |
| Gender | |  |  |  |  |  |  |  |  |  |  |
|  | Male | 159 (70.7) | 42 (35.9) | 38.4 | <.001 | 4.30 | 88 (61.1) | 18 (35.3) | 10.11 | <.001 | 2.88 |
|  | Female | 66 (29.3) | 75 (64.1) |  |  |  | 56 (38.9) | 33 (64.7) |  |  |  |
| ICD-10 SUD Alcohol | |  |  |  |  |  |  |  |  |  |  |
|  | Harmful use | 53 (36.6) | 26 (39.4) | 0.16 | .692 |  | 43 (40.6) | 13 (43.3) | 0.07 | .786 |  |
|  | Dependence | 18 (12.4) | 15 (22.7) | 3.66 | .056 |  | 11 (10.4) | 8 (26.7) | 5.16 | .023 | 3.14 |
|  | Any | 73 (45.6) | 48 (59.3) | 3.99 | .046 | 1.73 | 57(48.3) | 23 (59.0) | 1.24 | .248 |  |
| ICD-10 SUD Cannabinoids | |  |  |  |  |  |  |  |  |  |  |
|  | Harmful use | 59 (40.7) | 30 (45.5) | 0.42 | .516 |  | 52 (49.1) | 11 (36.7) | 1.44 | .230 |  |
|  | Dependence | 60 (41.1) | 25 (37.9) | 0.23 | .631 |  | 39 (36.8) | 12 (40.0) | 0.10 | .749 |  |
|  | Any | 126 (78.8) | 63 (77.8) | 0.03 | .493 |  | 98 (83.1) | 25 (64.1) | 6.20 | .013 | 0.36 |
| ICD-10 SUD Stimulants | |  |  |  |  |  |  |  |  |  |  |
|  | Harmful use | 37 (25.5) | 17 (25.8) | 0.00 | .970 |  | 28 (25.0) | 11 (34.4) | 1.11 | .293 |  |
|  | Dependence | 20 (13.8) | 16 (24.2) | 3.50 | .061 |  | 15 (13.4) | 7 (21.9) | 1.38 | .240 |  |
|  | Any | 63 (39.4) | 36 (44.4) | 0.57 | .450 |  | 44 (37.3) | 18 (46.2) | 0.96 | .326 |  |
| Any co-occurring psychiatric disorder beside SUD | |  |  |  |  |  |  |  |  |  |  |
|  | No | 100 (48.3) | 55 (51.9) | 0.36 | .549 |  | 74 (51.4) | 27 (54.0) | 0.10 | .750 |  |
|  | Yes | 107 (51.7) | 51 (48.1) |  |  |  | 70 (48.6) | 23 (46.0) |  |  |  |
| Physical abuse | |  |  |  |  |  |  |  |  |  |  |
|  | No | 179 (87.3) | 83 (79.0) | 3.36 | .057 |  | 89 (84.8) | 29(76.3) | 1.38 | .240 |  |
|  | Yes | 26 (12.7) |  |  |  |  | 16(15.2) | 9 (23.7) |  |  |  |
| Family violence | |  |  |  |  |  |  |  |  |  |  |
|  | No | 180 (87.0) | 84 (80.8) | 2.06 | .151 |  | 86 (79.6) | 34 (89.5) | 1.86 | .173 |  |
|  | Yes | 27 (13.0) | 20 (19.2) |  |  |  | 22 (20.4) | 4 (10.5) |  |  |  |
| Community violence | |  |  |  |  |  |  |  |  |  |  |
|  | No | 172 (83.1) | 74 (72.5) | 4.67 | .031 | 1.86 | 87 (81.3) | 27 (73.0) | 1.16 | .282 |  |
|  | Yes | 35 (16.9) | 28 (27.5) |  |  |  | 20 (18.7) | 10 (27.0) |  |  |  |
| Sexual abuse | |  |  |  |  |  |  |  |  |  |  |
|  | No | 184 (88.9) | 63 (60.0) | 35.25 | <.001 | 5.33 | 90 (84.9) | 21 (55.3) | 13.91 | <.001 | 4.55 |
|  | Yes | 23 (11.1) | 42 (40.0) |  |  |  | 16 (15.1) | 12 (44.7) |  |  |  |
| Physical neglect | |  |  |  |  |  |  |  |  |  |  |
|  | No | 180 (91.8) | 85 (84.2) | 4.09 | .043 | 2.12 | 87 (87.9) | 29 (78.4) | 1.94 | .164 |  |
|  | Yes | 16 (8.2) | 16 (15.8) |  |  |  | 12 (12.1) | 8 (21.6) |  |  |  |

Note. SUD are displayed if >10% in the total sample; The *n* for the comparisons may differ due to data availability

**Supplementary Table 3**

Logistic regression of the direct effect and interaction of gender and cannabis use disorders on suicidality

| **Suicidality** | **Variable** | **Regression Coefficient *B*** | ***SD*** | **Wald statistics (df)** | ***p*** |
| --- | --- | --- | --- | --- | --- |
|  |  |  |  |  |  |
| Suicidal ideation  (*n* = 240) | Gender | 0.39 | 0.60 | 0.43 (1) | .511 |
|  | ICD-10 F12 Any cannabis use disorder | -0.74 | 0.52 | 2.07 (1) | .150 |
|  | Interaction Gender*Any cannabis use disorder | 1.537 | 0.69 | 5.01 (1) | .025 |
|  | Constant | -0.83 | 0.45 | 3.33 (1) | .068 |
| Suicidal behavior  (*n* = 136) | Gender | 0.27 | 0.53 | 0.26 (1) | .611 |
|  | ICD-10 F12.2 Cannabis dependence | -0.83 | 0.71 | 1.36 (1) | .244 |
|  | Interaction Gender*Cannabis Dependance | 1.89 | 0.93 | 4.16 (1) | .041 |
|  | Constant | -1.44 | 0.37 | 15.10 (1) | <.001 |

*Supplementary Figure 1.* Interaction between gender and cannabis use disorders and suicidal ideation (*n* = 240, left) and suicidal behavior (*n* = 136, right)
